# Endoscopic papillectomy of major papilla lesions: Single tertiary care center experience

**DOI:** 10.1055/a-2663-6291

**Published:** 2025-08-14

**Authors:** Gaurav Suryawanshi, Mohamed Abdallah, Guru Trikudanathan, Stuart K Amateau, Shawn Mallery, Martin Freeman, Nabeel Azeem

**Affiliations:** 1 5635 Gastroenterology, Hepatology and Nutrition, University of Minnesota Twin Cities, Minneapolis, United States; 2 5635 Gastroenterology, Hepatology and Nutrition, University of Minnesota Twin Cities, Minneapolis, United States

**Keywords:** Endoscopic ultrasonography, Subepithelial lesions, GI surgery, GI Pathology, Tissue diagnosis, Pancreatobiliary (ERCP/PTCD), Diagnostic ERC

## Abstract

**Background and study aims:**

Endoscopic papillectomy (EP) is an effective endoscopic modality for managing ampullary lesions. This study aimed to evaluate predictors for recurrence and adverse events (AEs) in patients who underwent EP for major papilla lesions.

**Patients and methods:**

This was a retrospective analysis of all patients who underwent endoscopic snare papillectomy for a major papilla lesion between January 2006 and December 2021. We assessed multiple patient- and procedure-related variables to identify risk factors related to post-EP AEs and lesion recurrence using both univariate and multivariate analysis. In addition, we compared baseline characteristics and outcomes in patients with familial adenomatous polyposis (FAP) vs. sporadic ampullary lesions (SALs).

**Results:**

Fifty-one patients (11 FAP) were included in the final analysis. Recurrence was seen in 17 patients (37.0%) among those who followed up after technical success. Complete histological (R0) resection was the only factor associated with no recurrence following EP (odds ratio [OR] 5.4, 95% confidence interval [CI] 1.4–20.8, *P* = 0.014). PEP was associated with delayed bleeding (OR 7.5, 95% CI 1.2–46.1, *P* = 0.03). In FAP vs. SAL, lesion size was smaller in FAP: 10 mm (interquartile range 6–15 mm) vs. 15 mm (12–21 mm, *P* = 0.03) and the en-bloc resection rate was higher (100 vs. 67.5%, *P* = 0.01). Although the recurrence rate was higher in FAP vs. SAL (55.6 vs. 32.4%, *P* = 0.2), this was not statistically significant. Rates of AEs were similar.

**Conclusions:**

R0 resection was associated with reduced risk of recurrence whereas delayed bleeding after EP is associated with an increased risk of developing PEP. EP is safe and effective for removing ampullary lesions irrespective of lesion type.

## Introduction

Endoscopic papillectomy (EP) refers to resection of the duodenal mucosa and submucosa, including all the anatomic attachments of the ampulla of Vater and the tissues around the bile and pancreatic ducts 1 . EP is performed mainly for ampullary adenomas without evidence of invasive carcinoma 2 . EP is also used for early ampullary carcinoma, piecemeal resection of large laterally spreading lesions with deep ductal extension, a total biopsy method for tumor staging in the setting of resection margin compromise, and resection of non-adenomatous ampullary lesions 3 4 5 6 7 .

Although EP has a better safety profile than total pancreaticoduodenectomy or transduodenal ampullectomy, the adverse event (AE) rate can be as high as 35% 8 9 . The most common AEs are post-procedural bleeding (5%-20%), acute pancreatitis (3%-20%), perforation (0%-8%), cholangitis (0%-7%), and papillary stenosis (0%-7%) 3 9 10 11 12 13 . Recurrence rates for ampullary adenomas range between 8% and 32.7% 4 8 10 11 12 13 14 . Some studies have identified few risks factors for recurrence, such as piecemeal resection, intraductal growth, and size of the lesion, whereas complete histological (R0) resection and en bloc resection technique were found to be associated with low risk of recurrence 3 10 12 15 16 . However, in a retrospective international multicenter cohort study of 259 patients and data from 5-year follow‑up, Fritze et al. could not identify any risk factors for recurrence 17 .

EP and its efficacy in clinical settings are of particular importance when treating and evaluating patients with familial adenomatous polyposis (FAP). Patients with FAP have an elevated risk of developing ampullary and periampullary lesions, which can be found in 30% to 70% of FAP patients. In addition, duodenal or periampullary adenocarcinoma is the second leading cause of death in patients with FAP after colorectal cancer 18 19 . EP is felt to be a safe and effective procedure for patients with FAP. Recurrence is felt to be a bit more prevalent in patients with FAP compared with sporadic lesions with previous studies showing a recurrence rate of 14% to 58% 20 21 .

In this study, we aimed to retrospectively evaluate risk factors for recurrence and AEs following EP of ampullary lesions. In addition, we separately analyzed sporadic lesions from those associated with FAP and compared the resulting outcomes.

## Patients and methods

This study was a retrospective analysis of all consecutive patients who underwent EP for a major papilla lesion between January 2006 and December 2021 at a single institution. Patients who underwent endoscopic snare papillectomy with at least 6 months of follow-up were included in the analysis. If patients were found to have periampullary or duodenal lesions on endoscopy they were not included in this analysis. The study was approved by the Institutional Review Board at the University of Minnesota Medical Center.

Pre-procedural, procedural, and post-procedural data for all patients were collected. Pre-procedural data included age, gender, size of the ampullary adenoma, clinical presentation, presence of FAP syndrome, histology of pre-procedural biopsy, anticoagulation/antiplatelet therapy, and endoscopic ultrasound (EUS) evaluation for ductal invasion and regional extension. Procedural data included intraductal extension, presence of pancreas divisum, diverticulum, method of resection, argon plasma coagulation (APC) after resection, en bloc or piecemeal resection, need for submucosal lifting, technical success, biliary and pancreatic sphincterotomy, pancreatic stent placement, and biliary stent placement. Post-procedural data included histology of the endoscopic resection specimen, complete histological (R0) resection, number of procedures to achieve a complete resection, AEs, recurrence, histology of recurrence, management of recurrence, time to recurrence, and follow up. Lymph nodes were not assessed during EUS evaluation nor were they sampled, and thus, these data were not collected.

### Definitions

Technical success was defined as complete endoscopic resection as deemed at the time of the procedure. Recurrence was defined as any lesion identified on follow-up endoscopy after technical success was achieved as determined by the performing endoscopist. Intraductal extension was defined as an intraductal elevated lesion continuous with the papillary tumor that could be identified during endoscopic retrograde cholangiopancreatography or EUS. Procedure-related AEs were graded based on the guidelines of the American Society for Gastrointestinal Endoscopy 18 . Delayed post procedural bleeding was defined as clinical evidence of gastrointestinal bleeding following completion of the procedure up to 30 days that required admission, blood transfusion, or need for endoscopic, angiographic, or surgical intervention. Pancreatitis was defined, according to the revised Atlanta criteria, as presence of two of the three following criteria: 1) new onset or worsening of upper abdominal pain; 2) elevation of pancreatic enzymes (amylase and/or lipase) ≥ 3 upper limit of normal; and 3) imaging suggestive for pancreatitis 19 . Perforation of the duodenum or distal bile duct was defined as endoscopic visualization of a perforation or leakage of contrast or free fluid and/or abscesses on computed tomography (CT) performed after intervention. Cholangitis was defined, according to the Tokyo Guidelines 2018, as systemic inflammation (fever, shaking chills or laboratory evidence of inflammatory response) and either clinical or laboratory evidence of cholestasis or evidence of cholestasis on biliary imaging 20 . Papillary stenosis was defined as cholestasis with a proven papillary stenosis at cholangiogram during follow-up or dilatation of the pancreatic duct. R0 resection was defined as complete en bloc resection with negative margins on histologic examination. En bloc resection was defined as removing the lesions “as a whole or all together,” whereas piecemeal resection was defined as removing the lesions “in pieces with multiple steps”. Patients with piecemeal resection had difficult to determine histologic margins, and thus, were not considered to have R0 resection.

### Outcomes

The primary outcome of the study was incidence of ampullary lesion recurrence after a technically successful EP. Secondary outcomes included the number of procedures needed to achieve technical success, AE rate, concordance of histology pre- and post-EP, and evaluation of factors related to recurrence. Finally, outcomes of patients with sporadic ampullary adenomas and patients with FAP were compared.

### Endoscopic papillectomy procedure

**Fig. 1:**
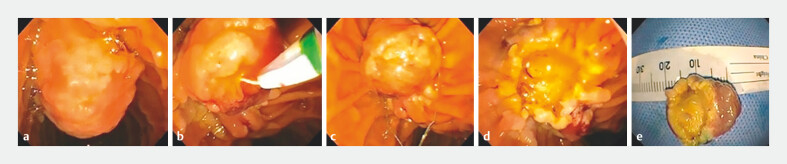
Endoscopic papillectomy (EP) of an ampullary adenoma. **a** Duodenoscopy showing a lesion at the duodenal ampulla. **b** Sphincterotome used to cannulate the major duodenal papilla. **c, d** En bloc resection of an ampullary mass. **e** Gross specimen of an ampullary adenoma following EP (Images courtesy of Dr. Nabeel Azeem, University of Minnesota).

All procedures were performed under fluoroscopic guidance with a therapeutic duodenoscope (TJF-Q180B; Olympus America, Center Valley, Pennsylvania, United States) with a 4.2-mm therapeutic working channel were used for all procedures. EP was performed by a team of five endoscopists with at least 3 years’ experience in pancreaticobiliary endoscopy. After identification of the lesion, cannulation of both biliary and pancreatic ducts was attempted, and if achieved, a cholangiogram/pancreatogram was obtained to delineate anatomy. Sometimes a biliary and pancreatic sphincterotomy was performed prior to resection. Snare resection was performed with the following settings: ERBE model VIO 300, ENDOCUT Q setting effect 3, cutting duration 1, and interval 4 (ERBE, Tübingen, Germany) ( Fig. 1 ). All procedures were performed under general anesthesia. No resected lesions were lost and all patients had final pathology.

### Follow-up

A first endoscopic evaluation was performed 4 to 6 weeks after the procedure with retrieval of any stents sometimes with systematic biopsies or, in case of residual/remnant adenoma, with a complementary resection on a further check 4 to 6 weeks later in the same manner. If patients were found to have residual/remnant adenoma then were not deemed to have technical success; seven patients required more than one procedure to achieve technical success. Follow-up was performed at 6 months and as needed thereafter to determine if patients had recurrence after technical success as defined above. Follow up included endoscopic examination and with or without biopsies of suspicious lesions. APC and intraductal radiofrequency ablation (RFA) were also utilized to clear residual tissue in certain cases.

### Statistical analysis

Baseline characteristics were reported as mean ± standard deviation (SD) or median (interquartile range [IQR]) for continuous variables and absolute numbers or percentages for categorical variables. For bivariate analysis, two-sample *t* -tests were used for normally distributed continuous variables and reported as (mean ± SD). Wilcoxon rank-sum tests were used for skewed continuous variables and reported as [median (interquartile ranges)], and chi-square tests were used for categorical variables. For multivariate analysis we picked the most clinically relevant variables and ran a logistic regression model to define independent predictors. *P* < 0.05 was considered statistically significant. Statistical analysis was performed using JMP, Version 15. SAS Institute Inc., Cary, North Carolina, United States, 1989–2019.

## Results

### Patient and lesion characteristics

**Fig. 2:**
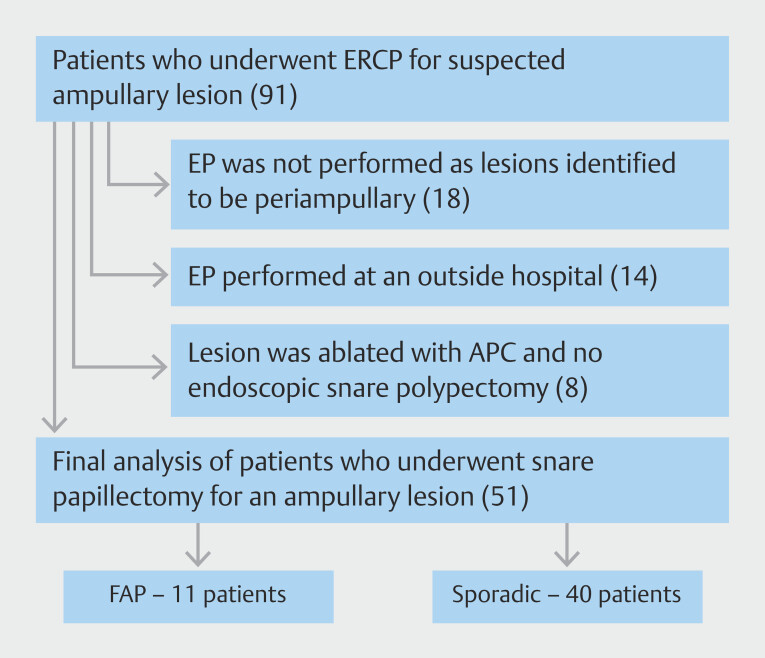
Flowchart of patients included in and excluded from study.

In the examined cohort, we reviewed a total of 91 patients with suspected ampullary lesions ( Fig. 2 ). Fifty-one patients underwent EP for ampullary lesions and were included in the final analysis. If patients were found to have periampullary or duodenal lesions on endoscopy, they were not included in this analysis. Baseline characteristics are summarized in ( Table 1 ).

**Table:** **Table 1** Patient, lesion, and procedure characteristics.

	**Total (n = 51)**	**FAP (n = 11, 21.6%)**	**Sporadic (n = 40, 78.4%)**	**P value**
Male, n (%)	29 (56.8)	4 (36.4)	25 (62.5)	0.12
Age, year, median (IQR)	65 (56–76)	35 (30–63)	70 (61–76.8)	< 0.001
Symptoms prior to EP, n (%)	22 (43.1)	2 (18.2)	25 (62.5)	< 0.001
Histology obtained prior to EP, n (%)	40 (78.4)	6 (54.5)	34 (85.0)	0.04
EUS prior to EP, n (%)	37 (72.5)	4 (36.4)	33 (82.5)	0.005
Antiplatelet/anticoagulation use, n (%)	14 (27.5)	0 (0.0)	14 (35.0)	0.02
Iron deficiency anemia, n (%)	15 (29.4)	4 (36.4)	11 (27.5)	0.71
Lesion size, mm, median (IQR)	15 (10.8–20)	10 (6–15)	15 (12–21)	0.03
Duration of procedure, minutes, median (IQR)	79 (57.8–101.3)	58 (53–81)	83 (58–102)	0.34
Method of resection				0.01
En bloc, n (%)	38 (74.5)	11 (100.0)	27 (67.5)	
Piecemeal, n (%)	13 (25.5)	0 (0.0)	13 (32.5)	
Intraductal extension, n (%)	8 (15.7)	1 (9.1)	7 (17.5)	0.67
Duodenal diverticulum, n (%)	4 (7.8)	0 (0.0)	4 (10.0)	0.57
Altered postsurgical anatomy, n (%)	2 (3.9)	0 (0.0)	2 (5.00)	1.00
Pancreas divisum, n (%)	3 (5.9)	0 (0.0)	3 (7.5)	1.00
Biliary sphincterotomy, n (%)	40 (78.4)	9 (81.8)	31 (77.5)	1.00
Pancreatic sphincterotomy, n (%)	11 (21.6)	3 (27.3)	8 (20.0)	0.68
Submucosal lifting prior to EP, n (%)	7 (13.7)	1 (9.1)	6 (15.0)	1.00
Pancreatic stent after EP, n (%)	46 (90.2)	10 (90.9)	36 (90.0)	1.00
Biliary stent after EP, n (%)	45 (88.2)	9 (81.8)	36 (90.0)	0.60
Final Pathology				0.4
Adenoma, n (%)	39 (76.5)	10 (90.9)	29 (72.5)	
Adenoma with high-grade dysplasia, n (%)	5 (9.8)	0 (0.0)	5 (12.5)	
NET, n (%)	1 (2.0)	0 (0.0)	1 (2.5)	
Adenocarcinoma, n (%)	2 (3.9)	0 (0.0)	2 (5.0)	
Other (non-neoplastic), n (%)	4 (7.8)	1 (9.1)	3 (7.5)	
Complete histological resection (R0), n (%)	23 (45.1)	5 (45.5)	18 (45.0)	1.00
Concordance of pre- and post- EP histology, n (%) *	35 (87.5)	6 (100)	29 (85.3)	1.00
# of procedures to achieve technical success				0.32
One, n (%)	44 (86.3)	11 (100.0)	33 (82.5)	
Greater than one, n (%)	7 (13.7)	0 (0.0)	7 (17.5)	
Recurrence after technical success, n (%)	17 (33.3)	5 (55.6)	12 (32.4)	0.2
Overnight admission after EP, n (%)	24 (47.1)	7 (63.4)	17 (42.5)	0.31
ED visit within 30 days of index EP, n (%)	7 (13.7)	2 (18.2)	5 (12.5)	0.64
Post-ERCP pancreatitis, n (%)	7 (13.7)	3 (27.3)	4 (10.0)	0.16
Papillary stenosis, n (%)	2 (3.9)	0 (0.0)	2 (5.0)	1.00
Procedural bleeding requiring endoscopic clips, n (%)	9 (17.6)	2 (18.2)	7 (17.5)	1.00
Delayed bleeding, n (%)	7 (13.7)	2 (18.2)	5 (12.5)	0.64
ED, emergency department; EP, endoscopic papillectomy; ERCP, endoscopic retrograde cholangiopancreatography; FAP, familial adenomatous polyposis; IQR, interquartile range; NET, neuroendocrine tumor.				

Forty of the 51 patients (78.4%) had sporadic lesions whereas 11 patients (21.6%) had FAP. Twenty-nine patients in the overall cohort were male (57%). The overall median age was 65 years (IQR 56–76) with a statistically significant difference ( *P* < 0.001) between the two groups: 35 years (IQR 30–63) in FAP patients and 70 years (IQR 61–76.8) in patients with sporadic lesions.

Overall, 22 patients (43%) had symptoms attributed to an ampullary lesion (jaundice, abdominal pain, and/or pancreatitis) prior to their EP. There was a significant difference between patients with sporadic lesions versus those with FAP who had symptoms prior to EP (62.5 v 18.2%, *P* < 0.001). Forty patients (78.4%) had histology obtained prior to EP whereas 37 patients (72.5%) had an EUS prior to EP. There was a statistically significant difference ( *P* < 0.05) in the number of patients with sporadic lesions who underwent EUS (82.5 v 36.4%) and had histology obtained prior to EP (85.0 v 54.5%) compared with patients with FAP. Thirty-four patients (67%) had either a computed tomography scan or magnetic resonance imaging (MRI) on file within 6 months of their initial EP; need for imaging was up to the discretion of the performing endoscopist or outpatient gastroenterology provider.

Among all the patients who underwent EP, 14 (27.5%) were on antiplatelet and/or anticoagulation therapy prior to EP; all these patients were in the sporadic group. Fifteen patients (29.4%) had iron deficiency anemia with no statistically significant difference between those with FAP (36.4%) and sporadic lesions (27.5%). Overall median lesion size was 15 mm (IQR 10.8–20) with a statistically significant difference ( *P* < 0.05) in size between FAP (10 mm [IQR 6–15]) and patients with sporadic ampullary lesions (15 mm [IQR 12–21]).

### Procedure characteristics

Median length of an EP procedure was 79 minutes (IQR 57.8–101.3); FAP patients had significantly shorter procedures (58 minutes [IQR 53–81]) compared with patients with sporadic lesions (83 minutes [58–102]).

Of the 51 patients who underwent EP, 38 (74.5%) underwent en bloc snare resection of their lesion, whereas 13 (25.5%) underwent piecemeal resection. All 11 FAP patients underwent en bloc snare resection compared with 27 patients (67.5%) with sporadic lesions ( *P* < 0.01).

Overall, eight patients (15.7%) had intraductal extension, four patients (7.8%) had a duodenal diverticulum, two patients (3.9%) had altered post-surgical anatomy, and three patients (5.9%) had a pancreas divisum. Forty patients (78.4%) underwent biliary sphincterotomy during EP whereas 11 patients (21.6%) underwent pancreatic sphincterotomy. Among patients who underwent en bloc versus piecemeal resection, seven of 38 had pancreatic sphincterotomy and 30 of 38 had biliary sphincterotomy; however, these differences were not statistically significant. Among the patients that underwent either biliary or pancreatic sphincterotomy (40 patients), six (15%) had a history of sphincterotomy prior to papillectomy. Within that group of six patients, four of six had en bloc resection. However, there was no statistically significant difference in R0 resection, incidence of recurrence, incidence of post-ERCP pancreatitis, or delayed bleeding following initial EP among these patients who had a history of sphincterotomy prior to initial EP. Seven patients (13.7%, 1 FAP and 6 sporadic) had submucosal lifting prior to EP. Forty-six patients (90.2%) had a pancreatic duct (PD) stent placed after EP and 45 patients (88.2%) had a biliary stent placed after EP (Table 1). Among the 45 patients who underwent biliary stenting, only one patient had a history of biliary stricture prior to initial EP; zero patients had clinical signs of cholangitis. Of patients who did not have a PD stent, 9.8% had a pancreatic divisum as demonstrated by prior MRI/magnetic resonance cholangiopancreatography or EUS.

Final pathology showed adenoma in 39 of 51 patients (76.5%), adenoma with high-grade dysplasia (HGD) in five of 51 (9.8%), adenocarcinoma in two of 51 (3.9%), neuroendocrine tumor (NET) in one of 51 (2.0%), and other (non-neoplastic lesions) in four of 51 patients (7.8%). Ten of 11 FAP patients (90.9%) had adenoma on final pathology with the remaining patient having a non-neoplastic lesion. Twenty-nine sporadic patients (72.5%) had an adenoma, five patients (12.5%) had adenoma with HGD, two patients (5.0%) had adenocarcinoma, one patient (2.5%) had a NET, and three patients (7.5%) had non-neoplastic lesions. Both patients with adenocarcinoma were referred to surgery and other specialists for further management but both declined any medical, endoscopic, or surgical interventions. One patient passed away after 15 months following an adenocarcinoma diagnosis. Overall, one of 11 FAP patients (9.1%) and six of 40 sporadic patients (15%) had non-adenomatous lesions on final pathology. However, there was no statistically significant difference with the final pathology between patients with FAP versus sporadic lesions. Concordance of pre- and post- EP histology was seen in 35 of 40 patients overall who had both pre- and post- EP biopsies (87.5%) and there was no statistically significant difference in concordance between FAP vs. patients with sporadic lesions.

### Primary outcome

Technical success was achieved in 100% of patients. Complete histological resection (R0) was achieved in 23 patients (45.1%) with no statistically significant difference between the FAP (5/11, 45.5%) and sporadic (18/40, 45.0%) groups. Forty-four patients (86.3%) only required one procedure to achieve technical success; all 11 FAP patients needed only one procedure, whereas seven sporadic patients (17.5%) required more than one EP to achieve technical success because they were determined to have residual/remnant lesions on follow up after initial procedure. R0 was only classified after patients had been determined to have technical success by the managing endoscopist. Median follow up was 228 days (IQR 40–795) for the 46 patients who followed up after technical success.

Recurrence was seen in 17 of 51 patients (33.3%): Five FAP patients (55.6%) and 12 sporadic patients (32.4%) were shown to have a recurrence on follow-up endoscopy after technical success. There was no statistically significant difference between the two groups with regard to timing of recurrence ( *P* = 0.2). Average recurrence free survival was approximately 657 days for these 51 patients.

### Management of recurrence

Overall, in those patients who followed up and were found to have recurrence after technical success, 13 patients (76.5%) underwent endoscopic management, two (11.8%) were referred to surgery, and two (11.8%) declined intervention. Among the 13 patients who underwent endoscopic management one (7.7%) underwent cold snare resection, one (7.7%) underwent RFA treatment, six (46.2%) underwent APC treatment, three (23.1%) had APC and snare, one (7.7%) had APC and RFA, and one (7.7%) had snare and RFA. Furthermore, of these 13 patients who had an endoscopy completed, two were found to have evidence of recurrence on future endoscopic evaluations.

### Adverse events

Of the 51 patients who were included in our analysis, 17 (33.3%) were noted to have intraprocedural bleeding. Major bleeding requiring further intervention with hemostatic clips was seen in nine patients (17.6%). Two patients with FAP (18.2%) and seven patients with sporadic lesions (17.5%) had major bleeding requiring hemostatic clips. Seven patients (13.7%) met criteria for post-ERCP pancreatitis, of whom three of 11 were FAP patients (27.3%) and four of 40 were sporadic patients (10%). Papillary stenosis was seen in two patients (3.9%), both of whom were patients with sporadic lesions. Delayed bleeding, defined as bleeding within 30 days of index EP, was seen in seven patients (13.7%) overall (2 FAP and 5 sporadic). Four patients with delayed bleeding required a blood transfusion. No cholangitis was reported within 30 days of index EP. There was no statistically significant difference between FAP and patients with sporadic lesions with regard to intraprocedural bleeding, bleeding requiring endoscopic clipping, post-ERCP pancreatitis, and papillary stenosis.

Twenty-four patients (47.1%) were admitted for observation following their index papillectomy. The most common reason for admission was post-procedural monitoring (18 patients), followed by post-ERCP pancreatitis (5 patients) and abdominal pain (1 patient). Seven patients (13.7%) were seen in the Emergency Department within 30 days of index discharge, whereas six patients (12.0%) were readmitted within 30 days of index discharge. Most common reason for readmission was gastrointestinal bleeding (four patients) and pancreatitis (two patients).

### Predictive factors for recurrence and adverse events

Risk factors for recurrence after endoscopic papillectomy were evaluated using univariate and multivariate analysis. After multivariate analysis, R0 resection (OR = 22.53, 95% confidence interval 1.41–360.5, *P* = 0.03) was the only variable found to be a predictor of no recurrence ( Table 2 ). Age, gender, lesion size, procedure duration, intraductal extension, and resection modality were not significant predictors of recurrence after technical success. Four of eight patients (50%) with intraductal extension had recurrence after technical success; however, this was not statistically significant ( *P* = 0.4). In addition, although the recurrence rate was higher in FAP vs. SAL (55.6 vs. 32.4%, *P* = 0.2), this difference was not statistically significant.

**Table:** **Table 2** Predictors of recurrence after technical success.

**Variables**	**OR (95% CI)**	***P* value **
Predictors of recurrence after technical success		
Age > 75	6.45 (0.23–181.56)	0.27
Male gender	0.56 (0.06–5.13)	0.61
Lesion size > 20 mm	0.33 (0.03–3.72)	0.37
R0 resection	**22.53 (1.41 – 360.5)**	**0.03**
FAP v sporadic	0.59 (0.03 – 13.98)	0.75
Duration of procedures > 90 min	1.47 (0.09–24.36)	0.79
Intraductal extension	4.25 e-8 (0-)	0.99
En bloc resection	5.2 e-9 (0-)	0.99
Submucosal injection	5.1 e-8 (0-)	0.99
Pancreas divisum	5.1 e+13(0-)	0.99
Predictors of delayed bleeding		
Age > 75	0.9 (0.16–5.5)	0.94
Male gender	0.18 (0.02–1.6)	0.13
Antiplatelets/anticoagulation	0.9 (0.16–5.5)	0.94
Intraprocedural bleeding	1.3 (0.22–7.5)	0.77
Post-ERCP pancreatitis	**7.5 (1.2–46.1)**	**0.03**
Predictors of post-ERCP pancreatitis		
Age > 75	4.3 e-8 (0-)	0.99
Male gender	0.7 (0.1–4.8)	0.70
Pancreatic duct stenting	0.5 (.04–6.3)	0.60
Intraprocedural bleeding	0.7 (0.9–5.5)	0.73
Delayed bleeding	**14.3 (1.4–146.8)**	**0.03**
CI, confidence interval; ERCP, endoscopic retrograde cholangiopancreatography; FAP, familial adenomatous polyposis; OR, odds ratio.		

When analyzing the cohort for predictors of AEs, we looked specifically at delayed bleeding and post-ERCP pancreatitis. When looking at delayed bleeding, age, gender, use of antiplatelets and/or anticoagulation, and intraprocedural bleeding were not significantly associated with delayed bleeding; however, post-ERCP pancreatitis was associated with delayed bleeding (OR 7.5, 95% confidence interval 1.2–46.1, *P* = 0.03). Furthermore, when looking at post-ERCP pancreatitis, age, gender, intraprocedural bleeding, and PD stenting were not significantly associated with occurrence of pancreatitis; however, delayed bleeding was significantly associated as detailed above ( Table 2 ).

In patients with non-R0 resection compared with those with R0 resection, there was no statistically significant difference ( *P* > 0.05) in incidence of post-ERCP pancreatitis, delayed bleeding within 30 days of initial EP, post-procedural bleeding requiring endoscopic management, and readmission to the hospital within 30 days of initial EP.

## Discussion

Endoscopic papillectomy is now widely accepted as a safe and reliable alternative to surgical intervention for management of lesions in the major papilla, whether they are benign adenomas or even early adenocarcinoma in certain situations 7 . Various studies have been conducted assessing risk factors for recurrence and/or post-procedural AEs in patients undergoing endoscopic papillectomy; however, most of these studies had mixed results and are heterogenous 4 9 10 11 13 14 17 .

In this retrospective analysis of 51 patients who underwent EP for ampullary lesions, with a median duration of follow up of 228 days (IQR 40–795), the recurrence rate was 33.3%. R0 resection, defined as en bloc resection plus negative margins on histology, was the only variable associated with reduced risk of recurrence. PEP was associated with development of delayed bleeding after EP or potentially vice versa. Our results showed that factors such as lesion size, intraductal extension, and use of APC were not associated with recurrence. This is consistent with studies conducted by Ridtitid et al. and Cecinato et al 12 21 .

Recurrence is challenging to measure because there is no uniform definition of recurrence, and in clinical practice, length of follow-up differs, ranging from 6 months to more than 5 years 3 10 14 15 . Due to lack of uniformity in defined follow-up period in the literature, the recurrence rate after EP has been reported anywhere between 13% and 33% 15 17 . Moreover, in the FAP population, it is difficult to know if a patient is having recurrence of the primary lesion or a new metachronous lesion. Multiple potential risk factors for recurrence have been suggested including FAP, presence of intraductal extension, piecemeal resection, and a final diagnosis of malignancy 10 16 . In our analysis, complete R0 resection, defined as en bloc plus negative margins on histology, was the most significant predictor of preventing recurrence following initial technical success. When analyzed independently, en bloc and piecemeal resection did not have a significant association with recurrence; neither did presence of intraductal extension.

When looking at the two groups of patients with major papilla lesions (FAP v sporadic) we found that FAP patients in our cohort had a higher rate of recurrence (55.6% v 32.4%); however, this was not a statistically significant difference. Regardless, given the nature of the disease, FAP patients would be expected to have an elevated risk for recurrence, and thus, they should have close surveillance after their initial procedure. In our cohort, all FAP patients who had recurrence underwent repeat endoscopy.

Another important highlight of our study is more accurate staging with the post-EP histology compared with biopsy, especially in patients with sporadic lesions. In our cohort, we looked at concordance between pre- and post- EP histology. Of the 40 patients who had both pre- and post-EP histology data, 35 were concordant. Of the five patients who did not have concordance, four were upstaged from adenoma to either adenoma with HGD (3/4 patients) or adenocarcinoma (1/4 patients). These findings highlight the inadequate staging that can occur with biopsy alone.

When it comes to EP, multiple factors, including patient-, procedure-, or even endoscopist-related factors, have been proposed to contribute to development of post-EP AEs. Given the significant potential risks of EP, identifying risk factors is crucial in order to best select patients suited for EP and attempt to reduce incidence of post-procedural AEs. Previous EP-related studies report AE rates ranging anywhere from 8% to 35% 14 17 21 . In our analysis, we evaluated patients who developed AEs within 30 days post-procedure. Post-ERCP pancreatitis was seen in 13.7% of patients and delayed bleeding was also seen in 13.7% of patients. Looking more closely at those patients (46) who had PD stents placed during initial EP, six (13%) developed post-ERCP pancreatitis compared with one of five (20%) in those who did not have a PD stent placed. There was no statistically significant difference in incidence of post-ERCP pancreatitis among those who received PD stents versus those who did not during initial EP. Incidence of pancreatitis lies within the range of previous literature (2%-20%) 14 16 17 21 . Of the 51 patients, 24 (47.1%) were admitted post-procedurally for overnight observation and six (12.0%) were readmitted to the hospital within 30 days. The decision to admit post-procedurally for observation was one that was made based on clinical assessment of the patient and at the discretion of the performing endoscopist. There is no defined protocol for post-procedure observation admission as of yet at our center. Of the six patients who were readmitted, four were readmitted due to gastrointestinal bleeding and two due to pancreatitis. Interestingly, average (mean) time between procedure and time of bleeding presentation was 2.86 days, which highlights the fact that post-procedural bleeding typically occurs rather acutely, and patients should be educated about it upon discharge. In our cohort, patients with delayed bleeding were managed as typical upper gastrointestinal bleeding patients with IV proton pump inhibitors, NPO status, and a transfusion threshold of hemoglobin < 7 g/dL. The decision to undergo endoscopy depended on the stability of the patient and endoscopist discretion; fortunately, none of the patients in our cohort presented with a hemodynamically significant bleed.

Also of note is that post-ERCP pancreatitis and delayed bleeding often presented together. This may be either because bleeding and subsequent clot formation at the papillectomy site is leading to pancreatic stent/duct obstruction or nearby pancreatic inflammation precipitates bleeding at the papillectomy site. This suggests that interventions to minimize either AE might reduce both. Furthermore, when looking at the subset of patients admitted overnight for observation (24 patients) versus those who were not (27 patients), those who were monitored overnight had a similar incidence of delayed bleeding (12.5 v 14.8%, *P* = 1.00), need to obtain medical attention for abdominal pain (8.3 v 14.8%, *P* = 0.67), need for readmission within 30 days of EP (4.2 v 19.2%, *P* = 0.2), and incidence of post-ERCP pancreatitis (20.8 v 7.4%, *P* = 0.23). Although none of these factors were statistically significant, post-procedure monitoring may be beneficial for some patients (frail or patients with comorbidities) or patients expected to develop a post-procedure AE.

The single-center experience, retrospective nature of our study, and follow-up duration are a few of the limitations of this study. The recurrence rate in our study was 33.3%, which can be considered to be at the higher end of the range compared with most published studies. Furthermore, we analyzed a heterogenous group of patient populations, pathologies, and resections techniques, which may limit generalizability of our study findings. In addition, our cohort is small with our two groups being only 11 and 40, which does limit the strength of our statistics. Despite these limitations, our study emphasizes the importance of achieving complete histologic resection because it was found to be the only variable associated with a decreased risk of recurrence after EP.

## Conclusions

In conclusion, EP is safe and effective for removing ampullary lesions irrespective of lesions type. In addition, delayed bleeding occurs early post-EP and is associated with development of post-ERCP pancreatitis and vice versa. Further studies are needed to optimize outcomes and minimize AEs in patients undergoing EP.

## References

[1] De Palma GD Endoscopic papillectomy: indications, techniques, and results World J Gastroenterol 2014 20 1537 1543 10.3748/wjg.v20.i6.1537 24587629 PMC3925862

[2] Chathadi KV Khashab MA Acosta RD The role of endoscopy in ampullary and duodenal adenomas Gastrointest Endosc 2015 82 773 781 10.1016/j.gie.2015.06.027 26260385

[3] Bohnacker S Seitz U Nguyen D Endoscopic resection of benign tumors of the duodenal papilla without and with intraductal growth Gastrointest Endosc 2005 62 551 560 16185970 10.1016/j.gie.2005.04.053

[4] Klein A Qi Z Bahin FF Outcomes after endoscopic resection of large laterally spreading lesions of the papilla and conventional ampullary adenomas are equivalent Endoscopy 2018 50 972 983 10.1055/a-0587-5228 29768645

[5] Ogawa T Ito K Fujita N Endoscopic papillectomy as a method of total biopsy for possible early ampullary cancer Dig Endosc 2012 24 291 10.1111/j.1443-1661.2011.01214.x 22725127

[6] Yamamoto K Itoi T Sofuni A Expanding the indication of endoscopic papillectomy for T1a ampullary carcinoma Dig Endosc 2019 31 188 196 10.1111/den.13265 30161275

[7] Vanbiervliet G Strijker M Arvanitakis M Endoscopic management of ampullary tumors: European Society of Gastrointestinal Endoscopy (ESGE) Guideline Endoscopy 2021 53 429 448 10.1055/a-1397-3198 33728632

[8] Heise C Abou Ali E Hasenclever D Systematic review with meta-analysis: endoscopic and surgical resection for ampullary lesions J Clin Med 2020 9 3622 33182806 10.3390/jcm9113622 PMC7696506

[9] Napoleon B Gincul R Ponchon T Endoscopic papillectomy for early ampullary tumors: long-term results from a large multicenter prospective study Endoscopy 2014 46 127 134 24477368 10.1055/s-0034-1364875

[10] Irani S Arai A Ayub K Papillectomy for ampullary neoplasm: results of a single referral center over a 10-year period Gastrointest Endosc 2009 70 923 932 10.1016/j.gie.2009.04.015 19608181

[11] Kang SH Kim KH Kim TN Therapeutic outcomes of endoscopic papillectomy for ampullary neoplasms: retrospective analysis of a multicenter study BMC Gastroenterol 2017 17 69 10.1186/s12876-017-0626-5 28558658 PMC5450406

[12] Ridtitid W Tan D Schmidt SE Endoscopic papillectomy: risk factors for incomplete resection and recurrence during long-term follow-up Gastrointest Endosc 2014 79 289 296 24094466 10.1016/j.gie.2013.08.006 PMC4413454

[13] van der Wiel SE Poley JW Koch AD Endoscopic resection of advanced ampullary adenomas: a single-center 14-year retrospective cohort study Surg Endosc 2019 33 1180 1188 10.1007/s00464-018-6392-9 30167949 PMC6430826

[14] Lee R Huelsen A Gupta S Endoscopic ampullectomy for non-invasive ampullary lesions: a single-center 10-year retrospective cohort study Surg Endosc 2021 35 684 692 10.1007/s00464-020-07433-7 32215745

[15] Takahashi K Ozawa E Yasuda I Predictive factor of recurrence after endoscopic papillectomy for ampullary neoplasms J Hepatobiliary Pancreat Sci 2021 28 625 634 10.1002/jhbp.992 33999505

[16] Kawashima H Ohno E Ishikawa T Endoscopic papillectomy for ampullary adenoma and early adenocarcinoma: Analysis of factors related to treatment outcome and long-term prognosis Dig Endosc 2021 33 858 869 10.1111/den.13881 33107134

[17] Fritzsche JA Klein A Beekman MJ Endoscopic papillectomy; a retrospective international multicenter cohort study with long-term follow-up Surg Endosc 2021 35 6259 6267 10.1016/j.giec.2022.01.005 33159297 PMC8523407

[18] Nass KJ Zwager LW van der Vlugt M Novel classification for adverse events in GI endoscopy: the AGREE classification Gastrointest Endosc 2022 95 1078 1085 e8 34890695 10.1016/j.gie.2021.11.038

[19] Banks PA Bollen TL Dervenis C Classification of acute pancreatitis--2012: revision of the Atlanta classification and definitions by international consensus Gut 2013 62 102 111 10.1136/gutjnl-2012-302779 23100216

[20] Miura F Okamoto K Takada T Tokyo Guidelines 2018: initial management of acute biliary infection and flowchart for acute cholangitis J Hepatobiliary Pancreat Sci 2018 25 31 40 10.1002/jhbp.509 28941329

[21] Jiang L Chai N Li M Therapeutic outcomes and risk factors for complications of endoscopic papillectomy: A retrospective analysis of a single-center study Ther Clin Risk Manag 2021 17 531 541 10.2147/TCRM.S309103 34093018 PMC8169047

